# The Very Long Chain Fatty Acid (C_26_:25OH) Linked to the Lipid A Is Important for the Fitness of the Photosynthetic *Bradyrhizobium* Strain ORS278 and the Establishment of a Successful Symbiosis with *Aeschynomene* Legumes

**DOI:** 10.3389/fmicb.2017.01821

**Published:** 2017-09-21

**Authors:** Nicolas Busset, Flaviana Di Lorenzo, Angelo Palmigiano, Luisa Sturiale, Frederic Gressent, Joël Fardoux, Djamel Gully, Clémence Chaintreuil, Antonio Molinaro, Alba Silipo, Eric Giraud

**Affiliations:** ^1^Institut de Recherche pour le Développement, LSTM, UMR IRD, SupAgro, INRA, Université de Montpellier, CIRAD Montpellier, France; ^2^Dipartimento di Scienze Chimiche, Complesso Universitario Monte Sant’Angelo, Università di Napoli Federico II Naples, Italy; ^3^Istituto per i Polimeri, Compositi e Biomateriali IPCB, Consiglio Nazionale delle Ricerche Catania, Italy

**Keywords:** VLCFA, lipid A, *Bradyrhizobium*, acyltransferase, symbiosis, *Aeschynomene*

## Abstract

In rhizobium strains, the lipid A is modified by the addition of a very long-chain fatty acid (VLCFA) shown to play an important role in rigidification of the outer membrane, thereby facilitating their dual life cycle, outside and inside the plant. In *Bradyrhizobium* strains, the lipid A is more complex with the presence of at least two VLCFAs, one covalently linked to a hopanoid molecule, but the importance of these modifications is not well-understood. In this study, we identified a cluster of VLCFA genes in the photosynthetic *Bradyrhizobium* strain ORS278, which nodulates *Aeschynomene* plants in a Nod factor-independent process. We tried to mutate the different genes of the VLCFA gene cluster to prevent the synthesis of the VLCFAs, but only one mutant in the *lpxXL* gene encoding an acyltransferase was obtained. Structural analysis of the lipid A showed that LpxXL is involved in the transfer of the C_26_:25OH VLCFA to the lipid A but not in the one of the C30:29OH VLCFA which harbors the hopanoid molecule. Despite maintaining the second VLCFA, the ability of the mutant to cope with various stresses (low pH, high temperature, high osmolarity, and antimicrobial peptides) and to establish an efficient nitrogen-fixing symbiosis was drastically reduced. In parallel, we investigated whether the BRADO0045 gene, which encodes a putative acyltransferase displaying a weak identity with the apo-lipoprotein *N*-acyltransferase Lnt, could be involved in the transfer of the C_30_:29OH VLCFA to the lipid A. Although the mutant exhibited phenotypes similar to the *lpxXL* mutant, no difference in the lipid A structure was observed from that in the wild-type strain, indicating that this gene is not involved in the modification of lipid A. Our results advance our knowledge of the biosynthesis pathway and the role of VLCFAs-modified lipid A in free-living and symbiotic states of *Bradyrhizobium* strains.

## Introduction

Rhizobia are Gram-negative bacteria with two life styles, one in a free-living state in the soil where they have to cope with changing environmental conditions (hydric, acid, saline stresses, nutrient starvation, etc.) and the other in symbiosis with plants, inside an organ called a nodule, in which they reduce atmospheric nitrogen to ammonium for the benefit of the host plant. A simplistic view would be that inside the nodule, the bacteria benefit from a safe ecological niche with unlimited carbon and energy provided by the plant in exchange for ammonium. In fact, inside the host cells, bacteria also encounter stressful conditions imposed by the plant (low oxygen tension, low pH, hyperosmosis, and various oxidative stresses) (Gibson et al., 2008; Haag et al., 2013). In addition, in some host species, including in inverted repeat-lacking clade (IRLC) legumes or the *Aeschynomene* species, the bacteria have to cope with antimicrobial peptides called NCR, that are used by plants to control bacterial metabolism and can led to a marked change in the shape of the bacteria (Van de Velde et al., 2010; Czernic et al., 2015; Montiel et al., 2017; Wang et al., 2017). The life of the rhizobia therefore does not resemble that of a long peaceful river since they have to adapt to changing stressful conditions outside and inside the host plants.

The first barrier used by the bacteria against biotic and abiotic stresses is its outer membrane (OM). Lipopolysaccharides (LPSs) are major components of the OM of Gram-negative bacteria. These compounds have three components (i) the O-antigen side chain that is in direct contact with the host plant, (ii) the core oligosaccharide, and (iii) a glycolipid moiety named the lipid A that anchors the LPS to the OM. LPSs are known to play a central role in bacterial invasion and adaptation to the host environment (Lerouge and Vanderleyden, 2002; Raetz and Whitfield, 2002). The variability of the O-antigen region observed in most rhizobia species is assumed to be a strategy to modulate or suppress plant defense responses, thereby facilitating the establishment of the symbiosis (Kannenberg and Carlson, 2001; Gourion et al., 2015). In addition, the lipid A of rhizobia LPSs is characterized by the presence of a C_26_ to C_30_ very long-chain fatty acid (VLCFA) (Bhat et al., 1994; Raetz et al., 2007). A lipid A-linked VLCFA is also encountered in pathogenic or intracellular bacteria such as *Brucella* or *Legionella*, suggesting that this structure promotes intracellular life by increasing the stability of the membrane (Bhat et al., 1991; Zähringer et al., 1995; Lerouge and Vanderleyden, 2002; Becker et al., 2005). The biosynthesis of the VLCFA and its addition to the lipid A require a cluster of five genes found in all the bacteria that synthesize a VLCFA-modified lipid A (Ardissone et al., 2011). This region is composed of genes encoding an acyl carrier protein (*acpXL*), fatty acid elongation proteins (*fabF*1*XL* and *fabF2XL*), a beta-hydroxyacyl-acyl carrier protein (ACP) dehydratase (*fabZXL*) and an acyltransferase (*lpxXL*) (Ardissone et al., 2011; Brown et al., 2011).

Analysis of *acpXL* and *lpxXL* mutants of *Rhizobium leguminosarum* and *Sinorhizobium meliloti* showed that the VLCFA plays a major role in the resistance to various abiotic stresses (high osmolarity, detergents, and dessication) but also in the establishment of a functional symbiosis (Ferguson et al., 2005; Haag et al., 2009; Ardissone et al., 2011; Bourassa et al., 2017). The plants inoculated with the VLCFA mutants are less efficient than the WT to fix nitrogen and present a nodulation delay. In addition, the nodules elicited by the VLCFA mutants present various alterations such as a white color, a smaller size, a disorganization of the infected zone and an early senescence. Furthermore, some bacteroids of the VLCFA mutants strains are abnormally large and have aberrant forms indicating also an alteration of the bacteroid differentiation process (Ferguson et al., 2005; Haag et al., 2009; Ardissone et al., 2011; Bourassa et al., 2017). Interestingly, while mutations in *acpXL* and *lpxXL* completely abolished VLCFA attachment to lipid A, it is observed that the *acpXL* mutants of *R. leguminosarum* and *S. meliloti* are able to substitute the VLCFA with a C16:0 or C18:0; which is not the case for *lpxXL* mutants (Ferguson et al., 2005; Bourassa et al., 2017). This suggests that in the absence of AcpXL, LpxXL could transfer shorter chain to the lipid A.

**FIGURE 1:**
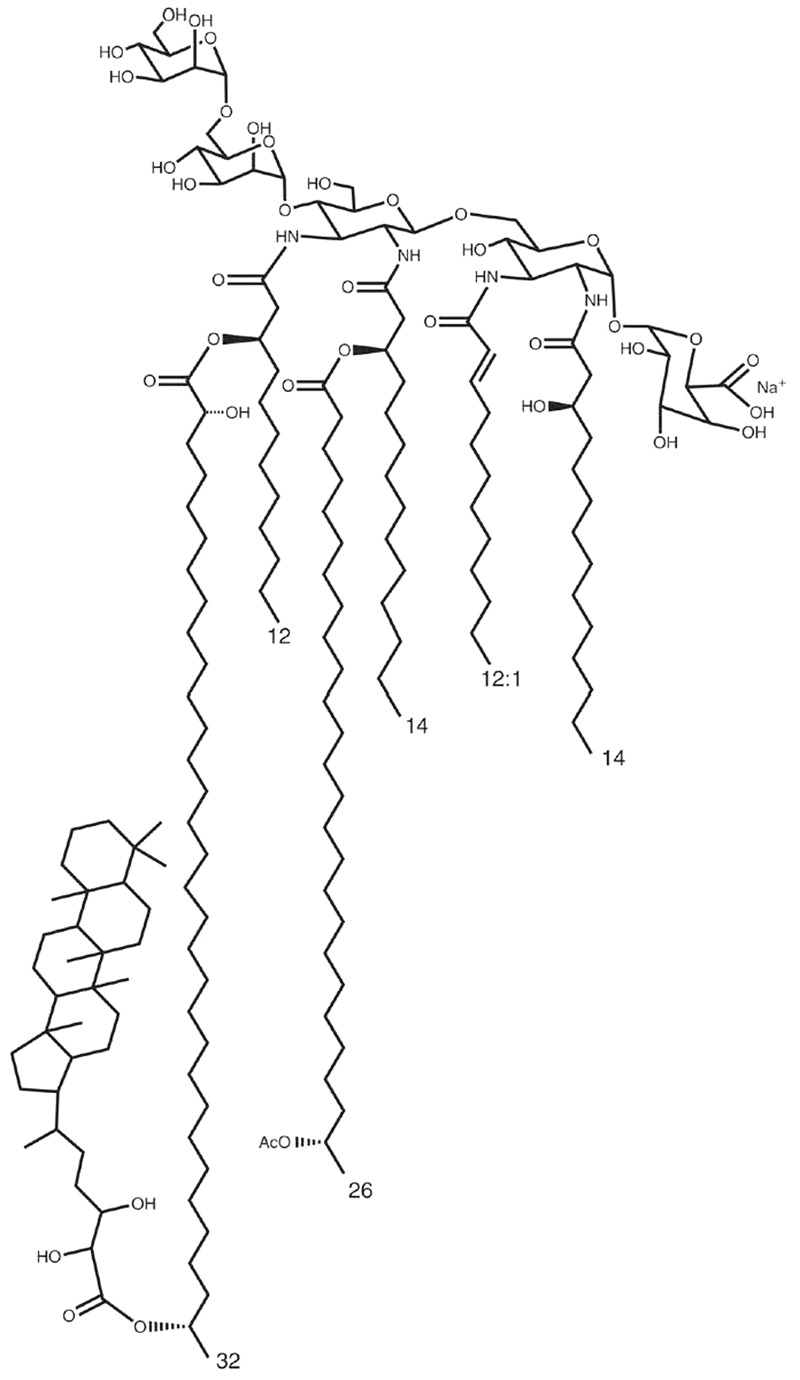
*Bradyrhizobium* BTAi1 lipid A structure (Silipo et al., 2014).

The OM of *Bradyrhizobium* strains has several peculiarities. First, unlike other rhizobia, hopanoid molecules are present in the membranes of all the *Bradyrhizobium* strains analyzed (Kannenberg et al., 1996). This family of compounds displays structural and functional similarities with eukaryotic sterols, such as cholesterol, and form an important class of membrane lipids that are widely distributed in diverse bacteria that reinforce the rigidity and stability of the OM (Ourisson et al., 1987; Kannenberg et al., 1996; Welander et al., 2009; Saenz et al., 2012). Second, structural analysis of the lipid A in various *Bradyrhizobium* strains revealed the occurrence of up to four VLCFAs that differ in their length and decoration (Choma and Komaniecka, 2011). Third, it has been reported that a hopanoid molecule can be covalently linked to the VLCFA(s) of the lipid A (**Figure 1**); this unusual lipid A structure was described for the first time in the photosynthetic *Bradyrhizobium* BTAi1 strain, named HoLA for Hopanoid-Lipid-A (Komaniecka et al., 2014; Silipo et al., 2014). Two recent studies reported that hopanoids play an important role in *Bradyrhizobium* strains by helping them to cope with various stresses in their two life styles (Silipo et al., 2014; Kulkarni et al., 2015). A hopanoid deficient mutant of the photosynthetic *Bradyrhizobium* BTAi1 strain, lacking a squalene hopene cyclase (Δ*shc*), displays increased sensitivity to stressful conditions and is unable to maintain chronic intracellular infection in *Aeschynomene* species (Silipo et al., 2014). Similarly, *hpnP* and *hpnH* mutants of *B. diazoefficiens* USDA110 affected in the synthesis of respectively methylated or extended (C35) hopanoids, displayed several disorders in both free-living and symbiotic states (Kulkarni et al., 2015). However, no study has been conducted on *Bradyrhizobium* strains to understand how the different VLCFAs linked to the lipid A are synthetized and to describe their respective roles.

**FIGURE 2:**
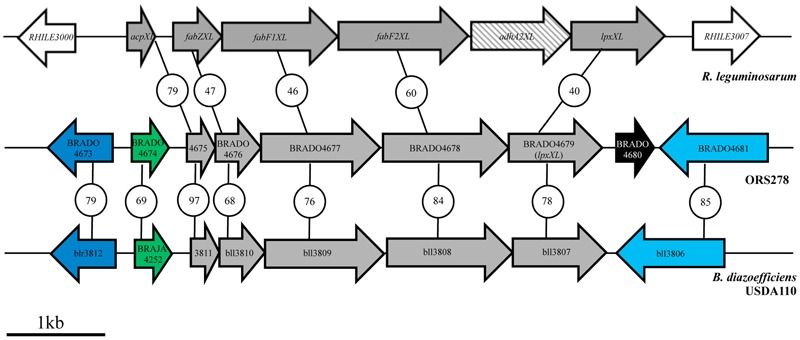
Identification of a gene cluster putatively involved in VLCFA biosynthesis in *Bradyrhizobium* strains by comparative genomic analysis with *R. leguminosarum*. The values in the circles represent the percentage of identity between the corresponding proteins. Genes in gray have been shown to be involved in VLCFA biosynthesis and their transfer to the lipid A in *R. leguminosarum*. The CDS in black is specific to photosynthetic *Bradyrhizobium* strains. Homologous genes are indicated by the same color. The genes are named according to their annotation in the published genomes (*R. leguminosarum* bv. *viciae* 3841, NC_008380; *Bradyrhizobium* sp. ORS 278, NC_009445.1; and *B. diazoefficiens* USDA110, NC_004463).

Interestingly, only one VLCFA gene cluster has been identified in the bradyrhizobial genomes (**Figure 2**). In this study, we investigated whether this cluster is responsible for the synthesis of the different VLCFAs linked to the lipid A using the photosynthetic *Bradyrhizobium* ORS278 strain as model. This strain has the unusual property of inducing the formation of nitrogen-fixing nodules on some tropical legumes of the *Aeschynomene* genus in the absence of the synthesis of Nod factor (Giraud et al., 2007). In addition, inside the host cell, bacteria undergo a drastic morphological change, suggesting important modifications of the bacterial cell wall (Bonaldi et al., 2011). This study focused on the effects of the mutations of the VLCFA gene cluster on the structure of the lipid A and on the free and symbiotic state of the bacteria.

## Materials and Methods

### Bacterial Strains and Growth Conditions

The bacteria used in this study are indicated in Supplementary Table S1. The *Bradyrhizobium* strain ORS278 and its derivatives mutants were grown in yeast extract mannitol (YM) medium at 34°C or in minimal medium buffered nodulation medium (BNM) (Podlešáková et al., 2013). *Escherichia coli* strains were grown in Luria-Bertani medium (LB) at 37°C. When required, the media were supplemented with kanamycin (100 μg ml^-1^) or spectinomycin (20 μg ml^-1^) or a mixture of the two (100 and 20 μg ml^-1^).

### Construction of ORS278 VLCFA Mutants

Standard molecular biology techniques were used for all cloning work. All the primers and plasmids used for cloning of DNA fragments are listed respectively in Supplementary Tables S1, S2. For the construction of *Bradyrhizobium* strain ORS278 insertional mutants, 300–400 base pair (bp) internal fragments were amplified by PCR and cloned into the plasmid pVO155-npt2-GFP (Okazaki et al., 2016). The constructions were first transferred into the *E*. *coli* S17-1 strain and then into ORS278 by mating as previously described (Giraud et al., 2010). For the construction of mutants by deletion mutagenesis, flanking regions of the genes were first amplified by PCR and then merged by overlap extension PCR. The overlap PCR fragment obtained was then digested by restriction enzymes corresponding to the restrictions sites of the primers (Supplementary Table S1) and was subsequently cloned in the *sacB* suicide pNPTS129 plasmid by ligation (Tsai and Alley, 2000). The spectinomycin resistance gene cassette from the pHRP315 vector (Parales and Harwood, 1993) was liberated by *Bam*HI digestion and was introduced between the upstream and downstream regions previously cloned in the pNPTS129 plasmid. The resulting plasmid was then transferred into ORS278 by biparental conjugation, as previously described (Giraud et al., 2010). Antibiotic selection was used to select single recombinant which were verified by PCR. Double recombinant clones were then obtained by growth on sucrose in the presence of spectinomycin but without the addition of kanamycin. Candidate clones were checked for the loss of kanamycin resistance from the pNPTS129 plasmid, and the deletion of the gene was verified by PCR.

### Lipid A Structure Analysis – MALDI MS and MS/MS

Lipopolysaccharide was isolated and purified from the ORS278 WT strain and the *lpxXl*, BRADO4680 and BRADO0045 mutants, as previously described, and the lipid A isolated through a mild acid hydrolysis (Silipo et al., 2014). Like for MS data, the lipid A was analyzed using a 4800 MALDI MS/MS (ABSciex) mass spectrometer equipped with a Nd:YAG laser at a wavelength (λ) of 355 nm with a 500-ps pulse and a 200 Hz firing rate. External calibrations were performed using an ABSciex calibration mixture, allowing mass accuracies close to 55 ppm. An aliquot of bacterial pellets was also subjected to a micro-extraction procedure to obtain the lipid A moiety, as described by El Hamidi et al. (2005), and analyzed in an ABSCIEX TOF/TOF^TM^ 5800 Applied Biosystems mass spectrometer equipped with an Nd:YLF laser with a λ of 345 nm, a <500-ps pulse length and a repetition rate of up to 1000 Hz. All mass spectra were acquired either in negative (not shown) and positive polarity. In the MS experiments, each spectrum resulted from the accumulation of 1,500 laser shots, whereas 5,000–7,000 shots were summed for the MS/MS data acquisitions (Spina et al., 2000; Sturiale et al., 2011). Samples were dissolved in CHCl_3_/CH_3_OH (50:50, v/v) at a concentration of 1 mg ml^-1^. Matrix solution was prepared by dissolving 2,4,6 trihydroxyacetophenone (THAP) in CH_3_OH/0.1% trifluoroacetic acid/CH_3_CN (7:2:1, by volume) at a concentration of 75 mg ml^-1^. One microliter of the sample/matrix solution (1:1, v/v) was deposited on the well plate and allowed to dry at room temperature (Leone et al., 2007).

### Growth Curves at Different Temperatures

To monitor growth in rich (YM) and minimal (BNM) media, cultures were inoculated at 10^-2^ dilution using YM-grown log-phase (OD_600_ = 0.5–0.7) WT or mutant strains. Growth was measured at OD_600_ using a Cary 50 Scan (Varian). Different incubation temperatures were tested (28, 34, and 37°C). Growth curves were performed in duplicate.

### Disk Diffusion Assays and NaCl Resistance Assay

The sensitivity to various abiotic stresses (SDS, H_2_O_2_, HCl, and NaCl) was assayed as previously described (Silipo et al., 2014). The experiments were conducted in triplicate for the *lpxXL* and BRADO4680 mutants and only once for the two BRADO0045 mutants.

### Resistance to Antibiotics

The minimum inhibitory concentration (MIC) of polymyxin B was determined as previously described (Kulkarni et al., 2015) by the Etest method using the disk diffusion assay (Biomérieux, Marcy-l’Etoile, France). The experiment was performed in triplicate.

### Plant Cultivation and Symbiotic Analysis

*Aeschynomene indica* and *A. evenia* seeds were surface sterilized, cultivated, and inoculated as previously described (Gully et al., 2016). For the nodulation and nitrogen fixation assay, 10 plants per condition were taken at 14 days post infection (dpi) to count the number of nodules on the roots and to analyze nitrogenase activity using an acetylene reduction assay (ARA) as previously described (Bonaldi et al., 2010).

### Cytological Analyses and Microscopy

Cytological analyses were conducted on 5–10 nodules originating from three different plants for each condition using the protocol described by Bonaldi et al. (2011). Confocal microscopy observations were carried out using a confocal laser-scanning microscope (Carl Zeiss LSM 700; Jena, Germany). Sections of ORS278 WT and BRADO4680 nodules were incubated for 20 min in live/dead staining solution [5 μM SYTO 9 and 30 μM propidium iodide (PI) in 50 mM Tris pH 7.0 buffer; Live/Dead BacLight, Invitrogen]. All the nodule sections were incubated for 15 min in 10 mM phosphate saline buffer (PBS) containing calcofluor white M2R (Sigma, Munich) at a final concentration of 0.01% (w/v) to stain the plant cell wall (Nagata and Takebe, 1970). Calcofluor was excited at 405 nm and emission signals were collected from 405 to 470 nm. For SYTO 9 or GFP and PI, an excitation wavelength of 488 and 555 nm was used with emission signal collected at 490–522 nm and 555–700 nm, respectively. Images were obtained using the ZEN 2008 software (Zeiss).

### Statistical Analysis

Statistical analysis was performed using XLSTAT version 2016.6 software. Differences between groups of samples were evaluated with the Tukey’s range test. Differences were considered statistically significant at a *P*-value < 0.01. The box plots were made in R 3.2.2 software. Results are shown as box plots. Each graph contains median, quartiles, and whiskers which show the last sample in [1st quartile -1.5^∗^IQR, 3rd quartile + 1.5^∗^ IQR] range. The free points on the graph represent the outliers samples.

### Accession Numbers

The GenBank accession numbers of BRADO4675 to BRADO4680 are respectively CAL78405 to CAL78410 and the one of BRADO0045 is CAL74017.

## Results

### *Bradyrhizobium* Strains Displayed Only One VLCFA Gene Cluster

A BLAST search of the ORS278 genome led to the identification of a single gene cluster containing several homologs of genes shown to be involved in VLCFA biosynthesis in the *R. leguminosarum* bv. *viciae* 3841 strain (**Figure 2**) (Bourassa et al., 2017). This gene cluster is composed of the CDS BRADO4675 to BRADO4679, which are homologs of *acpXL, fabZXL, fabF1XL, fabF2XL*, and *lpxXL* genes, respectively (**Figure 2**). Besides sharing a high level of identity (>40%) with the corresponding *R. leguminosarum* proteins, except for the absence of the a*dhA2XL* gene in ORS278, the organization of the genes is perfectly conserved between the two strains (**Figure 2**).

We also analyzed the distribution and the organization of the VLCFA genes in other photosynthetic (ORS285, BTAi1, *B. oligotrophicum* S58) and non-photosynthetic (*B. diazoefficiens* USDA110, *B. japonicum* USDA124, and *B. elkanii* USDA76) *Bradyrhizobium* strains. In all cases, only one homolog region was identified in which the genes displayed similar organization to that in ORS278 notably with the absence of the a*dhA2XL* homolog. However, the photosynthetic *Bradyrhizobium* strains differed from the non-photosynthetic ones by the presence, downstream of *lpxXL*, of a gene (BRADO4680) that codes for a protein of unknown function (**Figure 2**).

The identification of only one VLCFA gene cluster among the *Bradyrhizobium* genomes suggests that the genes present in this region might be sufficient for the synthesis and the attachment of the various VLCFAs that are linked to the lipid A in these bacteria.

### Lipid A of the *lpxXL* Mutant Lost the C_26_:25OH VLCFA, but Still Contains the C_30_:29OH VLCFA

To confirm that the identified region in the ORS278 strain is involved in the synthesis of VLCFAs and in their transfer to the lipid A, we tried to mutate the different genes present in this region, including BRADO4680. Different mutagenesis strategies were tested, deletion by double crossing over in *acpXL* and BRADO4680 and disruption of *fabZXL, fabF1XL, fabF2XL*, or *lpxXL* by insertion of the non-replicative plasmid pVO155-npt2-GFP in the corresponding coding region. Despite several attempts, only two mutants were obtained, one in the *lpxXL* homolog (BRADO4679) and the second in BRADO4680. The repeated failure to select mutants in *acpXL, fabZXL, fabF1XL*, or *fabF2XL* suggests that these four genes are essential because they are required for the synthesis of VLCFAs without which the bacteria cannot survive or grow under the conditions used to select the mutants. Conversely, the selection of mutants in BRADO4680 and *lpxXL* could result in functional redundancy, the absence of role, or in a specific role in the synthesis or the attachment of only one type of VLCFA to the lipid A.

**FIGURE 3:**
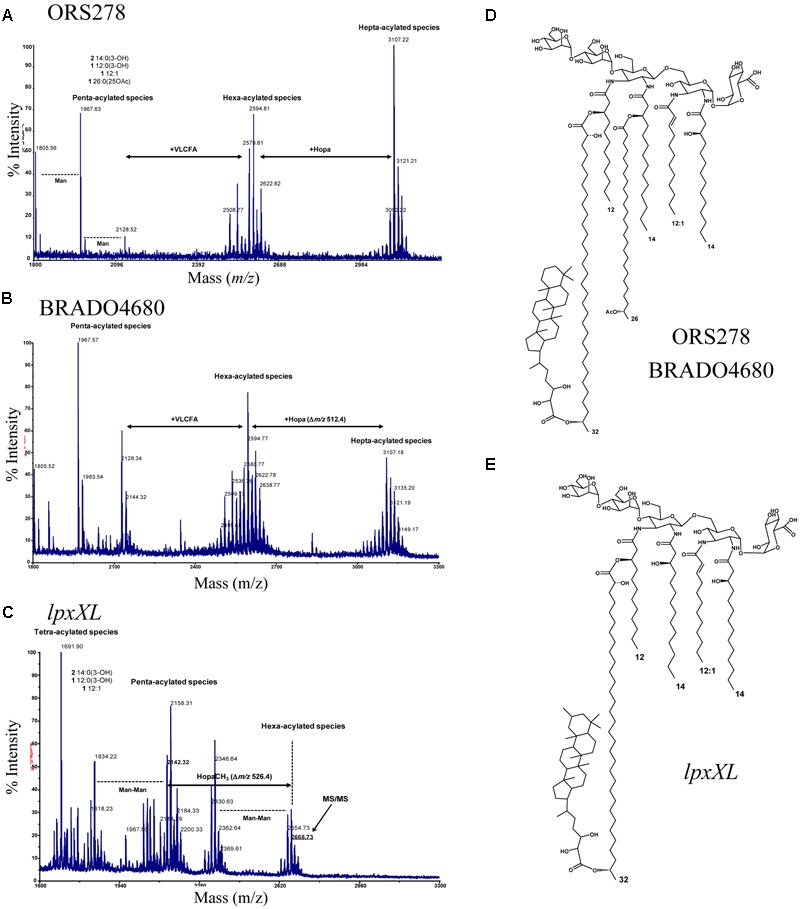
BRADO4679 (*lpxXL*) is involved in the transfer of the C_26_:25OH VLCFA to the lipid A of ORS278. **(A)** MALDI mass spectrum of lipid A from *Bradyrhizobium* ORS278; **(B)** MALDI MS analysis of lipid A from *Bradyrhizobium* ORS278Δ4680. The spectrum showed a series of intense sodiated molecular ions in the region between *m/z* 2400 and 2700, due to the hexa-acylated species carrying a VLCFA, and a further series of ions in the region between *m/z* 2900 and 3200 corresponding to hepta-acylated lipid A carrying a hopanoid unit (Δ*m/z* 512.4). The highest heterogeneity of lipid A from BRADO4680 is mainly due to variation of the chain length but not affecting the skeleton of the molecule. **(C)** MALDI mass spectrum of lipid A from *Bradyrhizobium lpxXL* mutant. The sodiated hexa-acylated lipid A, in the mass range between *m/z* 2500 and 3000, also comprised species carrying hopanoid moieties with an additional methyl-group (Δ*m/z* 526.4), as confirmed by the MS/MS analysis of the precursor ion at *m/z* 2668.73 (see Supplementary Figure S2). It is likely that methylation took place at C-2 of hopanoid residue, which frequently occurs under stress conditions and might play a key role in the permeability of the membrane. Proposed lipid A structures of ORS278 and BRADO4680 mutant **(D)** and *lpxXL* mutant **(E)**. The C_26_:25OH VLCFA was not observed in the *lpxXL* mutant of ORS278.

To investigate the impact of the BRADO4679 and BRADO4680 mutations on the synthesis of the VLCFAs, MALDI MS analyses were performed on the lipid A of the ORS278 WT and mutants strains. The mass spectrometry analysis of the WT lipid A showed a mixture of ion species which essentially varied by the acylation pattern, i.e., penta- and hexa-acylated species. All the lipid A species were stoichiometrically built up of a pentasaccharide backbone formed by a β-(1-6)-linked 2,3-diamino-2,3-di-deoxy-D-glucopyranose (DAG) carrying a GalpA residue on the vicinal DAG and an α-(1-6)-Manp disaccharide on the distal DAG residue. Indeed, the mass differences among neighboring ion species were due to acyl number and length of ester linked fatty acids (**Figure 3A**). The ion peak at m/z 2128.52 was established to be a penta-acylated sodiated lipid A carrying two 14:0 (3-OH), one 12:0 (3-OH), one 12:1 and one acetylated VLCFA, 26:0 (25-OAc). The most abundant sodiated hexa-acylated lipid A species (m/z 2594.81) further carried a secondary VLCFA, the 30:0 (2,29-2OH), in turn esterified, in the hepta-acylated species centered around *m/z* 3107.22, by the hopanoid moiety (**Figures 3A,D**). The ORS278 lipid A structure was therefore very similar to the one previously described for the BTAi1 strain with the presence of two VLCFAs, the longest of which harbored a hopanoid molecule (Silipo et al., 2014). The lipid A of the BRADO4680 mutant had a similar structure to that of the WT-strain (**Figures 3B,D**), showing that BRADO4680 is not involved in the modification of the lipid A.

In contrast, the lipid A of the *lpxXl* mutant showed differences in the acylation pattern. In fact, it was composed of a mixture of tetra-, penta-, and hexa-acylated species in which the 26:0 (25-OAc) VLCFA was absent (**Figures 3C,E** and Supplementary Figure S2), indicating that LpxXL is exclusively involved in the transfer of the C_26_:25OH VLCFA to the lipid A of ORS278.

### The C_26_:25OH VLCFA Lipid A Is Essential for the ORS278 Strain to Deal with Stresses in Free-Living Conditions

Very long-chain fatty acids have been shown to contribute to stress tolerance in free-living and symbiotic states, in diverse rhizobia like *R. leguminosarum* or *S. meliloti* (Becker et al., 2005; Gibson et al., 2008; Bourassa et al., 2017). To test the hypothesis that the removal of the C_26_:25OH of ORS278 lipid A reduces the ability of the strain to resist stressful conditions, we challenged *lpxXl* and BRADO4680 mutants with a variety of stressors that occur during the initiation and progression of symbiosis. In addition, the growth kinetics of the *lpxXl* and BRADO4680 mutants at various temperatures and their resistance to membrane destabilizer were quantified to determine if these mutations have an effect on the stability of the membrane.

**FIGURE 4:**
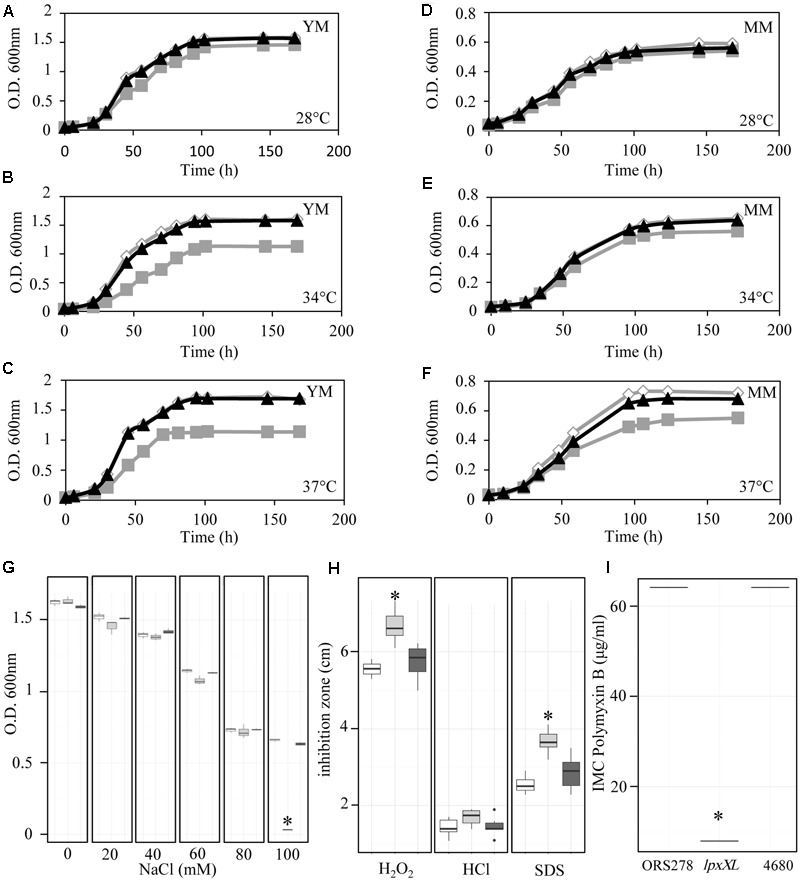
The lack of C_26_:25OH VLCFA affects the physiology of ORS278 in free-living state. **(A–F)** Growth kinetics of ORS278 (white), *lpxXL* (gray), and BRADO4680 (black) mutants in **(A–C)** YM medium and **(D–F)** Minimal medium at 28°C **(A–D)**, 34°C **(B–E)** and 37°C **(C–F)**, The experiment was carried out in duplicate. **(G)** Box plots representation of the NaCl resistance of ORS278 (white), *lpxXL* (gray) and BRADO4680 (dark gray) strains cultivated in rich medium (YM), at 34°C (*n* = 3). **(H)** Box plots representation of the hydrogen peroxide (H_2_O_2_), hydrogen chloride (HCl) and sodium dodecyl sulfate (SDS) resistance of ORS278 (white), *lpxXL* (gray) and BRADO4680 (dark gray) mutants, as determined by disk diffusion assays using 5 μl of 5.5 M H_2_O_2_, 2N HCl or 10% of SDS (*n* = 9). **(I)** Box plots representation of the polymyxin B resistance of ORS278 and the *lpxXL* and BRADO4680 mutants, determined by Etest (Etest^®^bioMérieux) on YM medium (*n* = 3). **(G–I)** ^∗^*P* < 0.01, by Tukey’s honestly significant difference test.

As shown in **Figures 4A,D**, the *lpxXl* mutant displayed similar growth to that of the WT strain at 28°C in both rich and minimal media. In contrast, at higher temperatures (34 and 37°C), the growth kinetics of the mutant were reduced compared with those of the WT strain (**Figures 4B,C,E,F**). These observations suggest a reduction in the stability of the membrane. In addition, disk diffusion assays showed that the *lpxXl* mutant was more sensitive to H_2_O_2_ and SDS than the WT strain (**Figure 4H**). The mutant was also more sensitive to osmotic stress than the WT strain, as evidenced by a reduction in its growth at a concentration of 100 mM of NaCl (**Figure 4G**). Because ORS278 is exposed to NCR-like peptides in *Aeschynomene* plants, we also tested the sensitivity of the mutants to polymyxin B which is a cationic peptide displaying similar effects than some NCRs on the alteration of the bacterial OM permeability (Mikuláss et al., 2016). Contrary to the *lpxXL* mutant of *R. leguminosarum* which is not affected in polymyxin B resistance (Bourassa et al., 2017), we observed that the *lpxXl* mutant of ORS278 displayed an eightfold lower MIC (8 μg/ml) for polymyxin B than the WT strain (64 μg/ml) (**Figure 4I**). The BRADO4680 mutant withstood all the stressors, as did the WT (**Figures 4A–I**). Together, these data suggest that the C_26_:25OH VLCFA linked to the lipid A of ORS278 plays an important role in the ability of this strain to cope with various stressful conditions by increasing the stability of the OM.

### The C_26_:25OH VLCFA Lipid A Is Essential for an Effective Symbiosis between the Photosynthetic *Bradyrhizobium* ORS278 Strain and *Aeschynomene* Plants

To investigate if the *lpxXl* and BRADO4680 mutations have an effect on the symbiotic properties of ORS278, we inoculated the WT and the two mutants strains on two *Aeschynomene* plants, *A. evenia* and *A*. *indica*, that can be nodulated in a Nod-independent manner by this strain.

**FIGURE 5:**
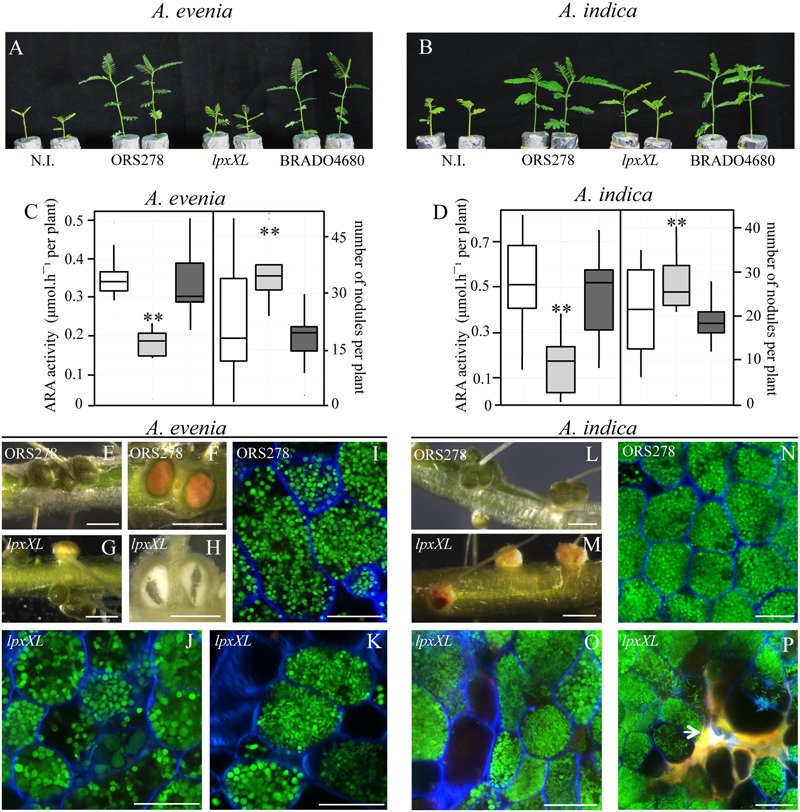
The *lpxXL* mutant of ORS278 is drastically impaired in symbiosis with *Aeschynomene* plants. Comparison of the growth of *A. evenia* **(A)** and *A. indica* **(B)** (aerial part), non-inoculated (N.I.) or inoculated with ORS278, *lpxXL* or BRADO4680 mutants. **(C,D)** Box plots representation of the quantification of acetylene reduction activity (ARA) and number of nodules per plant inoculated with ORS278 (white), *lpxXL* (gray) or BRADO4680 (dark gray) mutants in *A. evenia* **(C)** and *A. indica* **(D)**. The experiment was carried out in duplicate with 10 plants per condition; Tukey’s honestly significant difference test indicates a significant effect (^∗^*P* < 0.1; ^∗∗^*P* < 0.01). **(E–H,L,M)** Whole roots of *A. evenia* **(E–H)** and *A. indica* **(L,M)** inoculed with ORS278 **(E,F,L)** or the *lpxXL* mutant **(G,H,M)**; **(F,H)** cut nodules; scale bars: 2 mm. **(I–K,N–P)** Confocal microscopy observations of nodules from *A. evenia* **(I–K)** and *A. indica* **(N–P)** elicited by ORS278 **(I,N)**, or the *lpxXL* mutant **(J,K,O,P)**; scale bars: 20 μm. **(P)** The white arrow indicates a plant defense reaction.

No effect of the BRADO4680 mutation was detected on either *Aeschynomene* species (Supplementary Figure S1). In contrast, a clear effect of the *lpxXl* mutation was observed in both *Aeschynomene* species. Plants inoculated with this mutant, particularly *A. evenia*, displayed typical symptoms of nitrogen starvation, including leaf chlorosis and reduced plant growth at 14-dpi (**Figures 5A,B**). These observations were correlated with reduced nitrogenase activity, as estimated by the ARA compared to the WT strain, despite the fact that the *lpxXL* mutant led to a higher number of nodules per plant (**Figures 5C,D**). In addition, some nodules induced by the *lpxXL* mutant in both *A. evenia* and *A. indica* plants were yellowish instead of green, as observed for the WT nodules (**Figures 5E–G,L,M**). The absence of chloroplasts in the epidermal tissue of the *Aeschynomene* nodules is typically observed in mutants with altered nitrogen fixation, such as *nif* or hopanoid-minus mutants (Bonaldi et al., 2010; Silipo et al., 2014). Furthermore, some *A. evenia* nodules elicited by the *lpxXl* mutant were hollow suggesting a degradation of the tissue of the infection zone (**Figure 5H**), which is a sign of early senescence, as already reported in inefficient nitrogen-fixing mutants (Delmotte et al., 2014; Silipo et al., 2014). In *A. indica*, no hollow nodules were found, but brownish compounds that autofluorescent in the red spectrum (excitation, 488 nm; emission, 600–660 nm) were observed in some *lpxXl* nodules (**Figure 5P**). This is indicative of the accumulation of polyphenol compounds generally associated with plant defense reactions, as previously described in other legumes (Bourcy et al., 2013). Finally, cytological analysis performed by confocal microscopy showed that the process of bacteroid differentiation was altered in the *lpxXL* mutant, in both *A. evenia* and *A. indica* plants. In fact, in the *lpxXL* mutant, both undifferentiated bacteria and bacteroids were observed, which were not perfectly spherical and were of abnormal size, in contrast to the WT bacteroids (**Figures 5I–K,N–P**). As a WT reference, we used a tagged strain containing the pVO155-npt2-GFP plasmid inserted in the BRADO5083 gene which encodes a protein of unknown function. Previous studies did not reveal any particular symbiotic defect due to the plasmid insertion.

Taken together, these data indicate that the *lpxXL* mutation drastically alters the ability of the ORS278 strain to form an efficient symbiosis with *Aeschynomene* plants.

### Search for Another Acyltransferase Transferring the C_30_:29OH VLCFA to the Lipid A of ORS278

The fact that the lipid A of the *lpxXL* mutant still contains the C_30_:29OH VLCFA suggests that another acyltransferase exists that allows the specific transfer of this VLCFA to the lipid A. Sequence genome analysis of ORS278 did not enable identification of another homolog of *lpxXL*, suggesting that this acyltransferase should be strongly different from BRADO4679. Conversely, a search for genes annotated as containing an acyl transferase domain retrieved more than 40 candidates. This number was too high to envisage the systematic mutagenesis of all of them. On the other hand, a Tn5 mutant in one of them (BRADO0045) was previously described for its nitrogen fixing deficiency in *A. indica* (Bonaldi et al., 2010) suggesting that this gene plays an important role during symbiosis. This CDS has 42% identity with the ActA protein of *S. meliloti*, which has been shown to play an essential role in the acid tolerance of the bacteria (Tiwari et al., 1996), and 30% identity with the Apo-lipoprotein acyltransferase Lnt characterized in *E. coli*, which is involved in the maturation of lipoprotein (Gupta et al., 1993). Together, these different elements prompted us to examine in more detail the role of BRADO0045 in both the physiology and symbiotic properties of ORS278 and to explore the possibility of its direct involvement in the transfer of the C_30_:29OH VLCFA to the lipid A.

**FIGURE 6:**
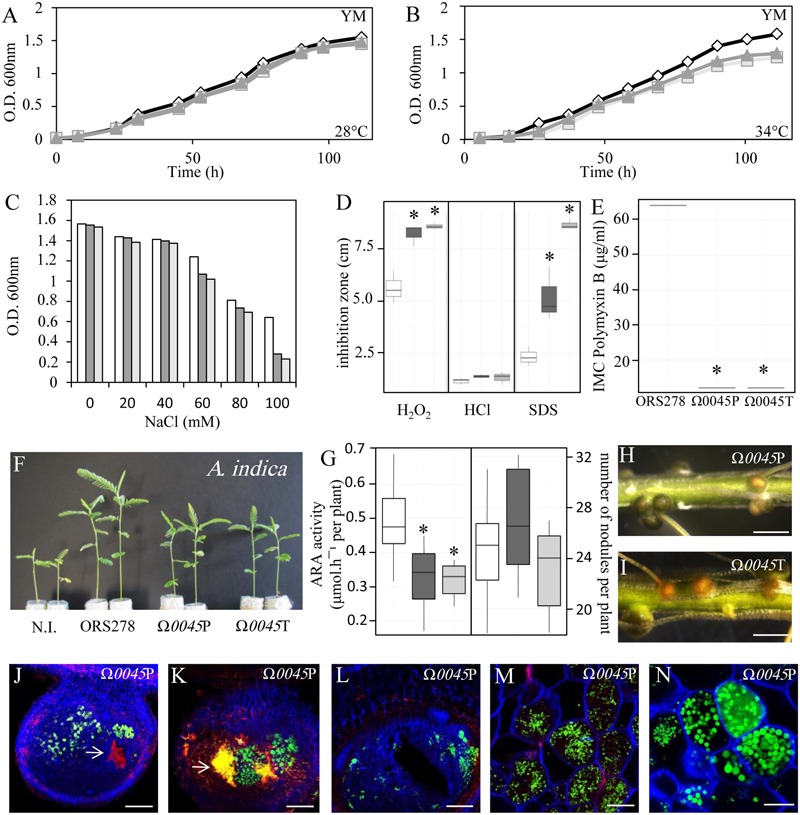
The BRADO0045 mutation affect both free-living and symbiotic states of ORS278. **(A–F)** Growth of ORS278 (white), Ω*0045*P (dark gray) and Ω*0045*T (gray) mutants in YM medium at 28°C **(A)** and 34°C **(B)** (*n* = 1). **(C)** NaCl resistance of ORS278 (white), Ω*0045*P (dark gray) and Ω*0045*T (gray) strains cultivated in rich medium (YM), at 34°C, *n* = 1. **(D)** Box plots representation of the Hydrogen peroxide (H_2_O_2_), hydrogen chloride (HCl) and sodium dodecyl sulfate (SDS) resistance of ORS278 (white), Ω*0045*P (dark gray) and Ω*0045*T (gray) mutants, as determined by disk diffusion assays using 5 μl of 5.5 M H_2_O_2_, 2N HCl or 10% of SDS (*n* = 9). **(E)** Box plots representation of the Polymyxin B resistance of ORS278 and the two Ω*0045*P and Ω*0045*T mutants, as determined by Etest (Etest^®^bioMérieux) on YM medium (*n* = 3); **(F)** Comparison of the growth of *A. indica* (aerial part), non-inoculated (N.I.) or inoculated with ORS278, Ω*0045*P or Ω*0045*T mutants. The experiment was carried out in duplicate with 10 plants per condition. **(G)** Box plots representation of the quantification of ARA and number of nodules per plant inoculated with ORS278 (white), Ω*0045*P (dark gray) or Ω*0045*T (gray) mutants in *A. indica*. Whole roots of *A. indica* inoculed with the Ω*0045*P mutant **(H)** or the Ω*0045*T mutant of ORS278 **(I)**; scale bars: 2 mm. **(J–N)** Nodule thin sections of *A. indica* elicited by the Ω*0045*P mutant and viewed by confocal microscopy; scale bars: 300 μm **(J–L)** and 20 μm **(M,N)**. **(J,K)** Whites arrows indicate plant defense reactions. **(D,E,G)** ^∗^*P* < 0.01, by Tukey’s honestly significant difference test.

To be sure that the phenotype reported for the Tn5 mutant corresponds to the inactivation of BRADO0045, we constructed a new insertional mutant in this CDS using the non-replicative plasmid pVO155-npt2-GFP. The ability of the two BRADO0045 mutants (Tn5 and pVO155) of ORS278, called Ω*0045*T and Ω*0045*P, respectively, to cope with various stresses was then analyzed as previously. Analysis of growth kinetics at 28 and 34°C showed that the two BRADO0045 mutants underwent alteration of their growth at the higher temperature, pointing to destabilization of the membrane, as observed in the *lpxXL* mutant (**Figures 6A,B**). In addition, the two mutants were found to be more sensitive to salt, oxidative and detergent stresses and to the polymyxin B (**Figures 6C–E**) than the WT strain. These results show that the mutation in BRADO0045 reduces the ability of ORS278 to cope with abiotic stresses in the same way as the *lpxXL* mutation.

To test if the mutations in BRADO0045 have an impact on the symbiotic properties of ORS278, *A. indica* plants were inoculated with the WT strain and the two mutants. At 14-dpi, we observed that the plants inoculated with the two mutants were smaller than the plants inoculated by the WT-strain (**Figure 6F**), which is correlated with lower nitrogenase activity (**Figure 6G**). As previously observed in the *lpxXL* mutant, some nodules elicited by the two BRADO0045 mutants were yellow and hollow (**Figures 6H,L**) and some displayed massive plant defense reactions (**Figures 6I–K**). In addition, cytological analysis showed that the intracellular bacteria were not differentiated or not perfectly spherical, and were of abnormal size, the same as observed in the *lpxXL* mutant (**Figures 6M,N**). The BRADO0045 protein thus appears to be essential for the establishment of an efficient symbiosis between ORS278 and *A. indica*.

To determine if this phenotype was due to alteration of the lipid A structure of ORS278, MALDI MS analyses were performed on the lipid A from ORS278 WT and from Ω*0045*P mutant. As can be seen in Supplementary Figure S3, the two spectra were very similar, showing that the effects of the BRADO0045 mutation on the phenotypic and symbiotic properties of ORS278 were not due to modification of the lipid A.

## Discussion

The fact that a VLCFA is attached to the lipid A of rhizobia was first described in *R. leguminosarum* bv. *trifolii* more than two decades ago (Hollingsworth and Carlson, 1989). Since this discovery, several studies have (i) highlighted the ubiquitous presence of a VLCFA in all the rhizobium lipid A studied, (ii) revealed the importance of this VLCFA in both the free and symbiotic life forms of several rhizobium species and (iii) characterized the genes involved in its biosynthesis (Bhat et al., 1991; Ferguson et al., 2005; Raetz et al., 2007; Haag et al., 2009; Ardissone et al., 2011; Bourassa et al., 2017). More recently, it was shown that the lipid A of several *Bradyrhizobium* strains differs in the presence of at least two VLCFAs, one of which can be linked to a hopanoid molecule (Choma and Komaniecka, 2011; Komaniecka et al., 2014; Silipo et al., 2014). However, nothing is known on the synthesis and the respective role of these VLCFAs.

In this study, we have shown that *Bradyrhizobium* genomes contain a gene cluster homologous to the one described in *R. leguminosarum* that enables the synthesis and the attachment of the VLCFA to the lipid A. In the *Bradyrhizobium* ORS278 strain, we succeeded in obtaining a mutant in only one gene of this cluster, the BRADO4679 gene homologous to *lpxXL* and showed that this mutation led to the suppression of only the C_26_:25OH VLCFA. This indicates that this gene encodes an acyltransferase which catalyzes the transfer of such a VLCFA to the lipid A but not the one of the C_30_:29OH VLCFA. Despite the fact that the mutant maintains this second VLCFA linked to the lipid A, it displays several phenotypes similar to those reported in *lpxXl* mutants in other rhizobia species, such as a higher sensitivity to various stresses (NaCl, H_2_0_2_, HCl) and alteration of the efficiency of the symbiosis (Ferguson et al., 2005; Haag et al., 2009; Ardissone et al., 2011). In particular, we observed that the *Aeschynomene* nodules elicited by the *lpxXL* mutant of ORS278 displayed several disorders: some nodules were yellowish and the bacteroids were malformed. Furthermore, some *A. indica* nodules accumulated autofluorescent brown compounds suggesting the induction of plant defense reactions while the central tissue of some *A. evenia* nodules was digested. This degradation of the symbiotic tissue is typically observed in senescing nodules such as described in *Medicago* or other legume species (Puppo et al., 2004). It would be interesting to confirm that the *LpxxL* mutant can induce plant defense reactions and early nodule degeneracy, respectively, on *A. indica* and *A. evenia*, by checking the expression of some senescence and pathogenesis-related genes. Nevertheless, *Aeschynomene* is just emerging as a new model legume and marker genes of these two cellular programs have to be first characterized for these two species. These severe phenotypes are probably related to the inability of the mutant to cope with the stresses found in the host cell (oxidative stresses, NCR peptides, acid stress) but also to perturbations of the LPS and/or the OM structure that could compromise the recognition of the bacteria by the plant which then reacts by the induction of a defense response or by triggering senescence. In agreement with this latter possibility, it has been recently identified in *Arabidopsis* a lectin S-domain receptor kinase that detects the lipid A moiety and modulates immune response to bacterial infection (Ranf et al., 2015). It is also important to note that other mutants which displayed some modifications in the structure of their cell wall, such as the DD-CPase1 mutant of ORS278 strain, which is altered in the level of reticulation of the peptidoglycan, or a hopanoid *minus* mutant of the BTAi1 strain, exhibited very similar phenotypes to the *lpxXL* mutant (Silipo et al., 2014; Gully et al., 2016). This suggests that the structural integrity of the bacterial cell envelope of which the attachment of the C_26_:25OH VLCFA to the lipid A is an important determinant, is essential to maintain chronic intracellular infection during symbiosis.

In this study, we did not succeed in mutating the *acpXL, fabZXL, fabF1XL*, or *fabF2XL* genes of the ORS278 strain. Taking into account the fact that an *lpxXL* mutant was obtained with the previously described features, it can be assumed that these four genes are involved in the synthesis of the two VLCFAs and that in the absence of their synthesis, the ORS278 strain cannot survive. In support of this last hypothesis, a Tn-seq-based study of *Rhodopseudomonas palustris* CGA009, a strain phylogenetically close to *Bradyrhizobium*, indicates that these genes are also essential in this bacteria (Pechter et al., 2015). In addition to this common pool of genes, we predict that at least three additional enzymes are necessary to achieve the complete synthesis of the lipid A in its most complex form: (i) an enzyme permitting elongation of the C_26_:25OH VLCFA to the C_30_:29OH VLCFA genes, (ii) an acyltransferase specific to the C_30_:29OH VLCFA allowing its transfer to the lipid A, and finally, (iii) an enzyme catalyzing the attachment of the C_35_-hopanoid to this VLCFA. The fact that a *shc* mutant of BTAi1 displayed a lipid A with the two VLCFAs but lacking the hopanoid moiety leads us to think that, first, the C_30_:29OH VLCFA is attached to the lipid A and, second, the hopanoid is attached to the C_30_:29OH VLCFA. Some genes of the lipid A biosynthesis pathway in *Bradyrhizobium* strains therefore remain to be identified. Their discovery will be a real challenge considering that their mutation could be lethal and that no other homolog of *lpxXL* has been identified in the *Bradyrhizobium* genomes.

In this context, we examined whether the BRADO0045, that encodes a putative acyltransferase and for which a Tn5 mutant has been reported to be affected in nitrogen fixation, can catalyze the transfer of the C_30_:29OH VLCFA to the lipid A. MALDI MS analyses of the lipid A clearly showed that BRADO0045 does not play this role since no structural difference was observed with the WT lipid A. On the other hand, the phenotypic properties of the BRADO0045 mutants are very similar to those of the *lpxXL* mutant indicating that the mutation of BRADO0045 would affect the structure of the cell envelope. The protein BRADO0045 has a low level of identity with the apo-lipoprotein *N*-acyltransferase Lnt of *E. coli*. This enzyme plays a role in the maturation of lipoproteins by catalyzing the attachment of a third acyl chain that enables their transfer to the OM by the Lol system in Gram-negative bacteria (Fukuda et al., 2002). It has been shown in *E. coli* that the mutation of *lnt* affects the properties of both the inner and OM by reducing the level of incorporation of lipoproteins in the OM and, consequently increasing it in the inner membrane, which is lethal in this bacterium (Robichon et al., 2005; Narita and Tokuda, 2011). It would therefore be interesting to compare the lipoprotein composition of the different membrane compartments in the BRADO0045 mutant and in the WT strain to check whether BRADO0045 corresponds to an apo-lipoprotein *N*-acyltransferase. It is also to note that the three downstream genes of BRADO0045 (BRADO0046 to BRADO0048) are in the same direction than BRADO0045 (see Supplementary Figure S4), we cannot therefore exclude the possibility that the phenotype observed for the BRADO0045 insertional mutants was due to polar effects.

Numerous studies have revealed an essential role for VLCFA-modified lipid A in bacteria with an intracellular lifestyle, whether they are pathogens or symbionts like rhizobia (Bhat et al., 1991; Zähringer et al., 1995; Becker et al., 2005). One may wonder why, unlike other rhizobia, *Bradyrhizobium* strains, which have similar lifestyles, have at least two VLCFAs linked to their lipid A instead of one and why a hopanoid molecule is covalently linked to this second VLCFA. It was previously shown by analysis of reconstituted liposomes using electron spin resonance (ESR) spectroscopy, that, thanks to their VLCFAs, HoLA molecules can span the whole OM by placing the hopanoid moiety in the inner leaflet, which results in a higher stabilization of the inner and the outer leaflets of the OM (Silipo et al., 2014). This rigidification of the OM might be a functional advantage by strengthening its barrier role, which could facilitate the survival of the bacteria under stress conditions. This could contribute to the ecological success of the *Bradyrhizobium* genus which nodulates the widest range of legume species and which is distributed worldwide but predominately in tropical areas and acid soils (Parker, 2015; Sprent et al., 2017).

## Author Contributions

NB, EG, AM, and AS conceived the experiments, NB, FDL, AP, LS, FG, JF, DG, and CC conducted the experiments; NB, EG, AM, and AS analyzed the results and wrote the paper.

## Conflict of Interest Statement

The authors declare that the research was conducted in the absence of any commercial or financial relationships that could be construed as a potential conflict of interest.
