# Supplementary material for: The Very Long Chain Fatty Acid (C26:25OH) Linked to the Lipid A Is Important for the Fitness of the Photosynthetic Bradyrhizobium Strain ORS278 and the Establishment of a Successful Symbiosis with Aeschynomene Legumes
Source: Front Microbiol. 2017 Sep 21;8:1821. doi: 10.3389/fmicb.2017.01821 (PMC5613085; doi:10.3389/fmicb.2017.01821)
Supplement: Supplementary file 1 [file Table_1.DOCX]

**Table S1: Strains and plasmids used in this study.**

| **Strains** | **References** |
| --- | --- |
| ORS278 | Giraud *et al*, 2007 |
| *E. coli* xl2-blue | Stratagene |
| *E. coli* S17-1 | Grohmann *et al*, 2003 |
| ORS278ΩO0045T | Bonaldi *et al*, 2010 |
| ORS278Ω*lpxXL* | Insertional mutant in BRADO4679 |
| ORS278Δ4680 | Deletion mutant of BRADO4680 |
| ORS278Ω0045P | Insertional mutant in BRADO0045 |
| **Plasmids** | **References** |
| pVO155-npt2-gfp | Okazaki *et al*, 2016 |
| pNPTS129 | Tsai and Alley, 2000 |

**Table S2: Primers used in this study.**

| **Primers** | **Sequences** | **Relevant characteristics** |
| --- | --- | --- |
| BRADO0045.f/BRADO0045.r | CGTCTCTCGAGTAGCGATGGCCCCGTTCAATG/  AGCGGTCTAGATAGCGCATAGCCGAACGTGTTC | Cloning of BRADO0045 fragment in pVO155-npt2-gfp after digestion of the PCR product by XhoI/XbaI |
| Up.BRADO4675.f/Up.BRADO4675.r  Down.BRADO4675.f/Down.BRADO4675.r | CTTTGTAGATCTCTGCGGCTGCGTGCTATGGAAAG/  TGGCGGGCGCAAGCTTCACTAAGCCTTTGATATTTTG  AGGCTTAGTGAAGCTTGCGCCCGCCATGCAGATCGAATAC/  CGCTGCCTCTAGACCATCTGGCGCTGTTCCTTCTTC | Cloning of flanking regions of BRADO4675 in the suicide plasmid pNPTS129 after digestion of the PCR product by BglII/Xba1 |
| BRADO4676.f/BRADO4676.r | ACCGCGTCGACGACCTCAAGCTGGACGAGAAGAC/  ACGGCTCTAGAGAACGGCATCCGCTCGAACTTC | Cloning of BRADO4676 (fabZ) fragment in pVO155-npt2-gfp after digestion of the PCR product by SalI/XbaI |
| BRADO4677.f/BRADO4677.r | AATCTCTCGAGGCAGATTCCGAAGAAGGAACAG/  CGCCATCTAGAGAGCCGCCGACCAGCGCGATGTC | Cloning of BRADO4677 (fabF1) fragment in pVO155-npt2-gfp after digestion of the PCR product by XhoI/XbaI |
| BRADO4678.f/BRADO4678.r | CTGGGGTCGACCAAGTCCGACAATTGGACCAAG/  GTGAGTCTAGAGAGCCCTTGGTACCGAATGTATC | Cloning of BRADO4678 (fabF2) fragment in pVO155-npt2-gfp after digestion of the PCR product by SalI/XbaI |
| BRADO4679f./BRADO4679.r | GCCGTCGTCGACCTGACGGTCGGCATGCTCAAG/  GGGCGTCTAGAGCCAGCGCCGGCATTTCCCAATTG | Cloning of BRADO4679 fragment in pVO155-npt2-gfp after digestion of the PCR product by XhoI/XbaI |
| Up.BRADO4680.f/Up.BRADO4680.r  Down.BRADO4680.f/Down.BRADO4680.r | TCGAGGGATCCCCGCGCACGCACGAGCTGTTTCAC/  GCCGGAGAGAAAGCTTTCGCGCTTGTCCTCCCTGGAGCTTC  ACAAGCGCGAAAGCTTTCTCTCCGGCGTCATTGCGAGGAGCAAC/  GGCGTGGGTACCGACAGCTGGGACATGGTGTTCAAG | Cloning of flanking regions of BRADO4680 in the suicide plasmid pNPTS129 after digestion of the PCR overlap product by BamHI/KpnI |
