# Supplementary figures and images for: The Very Long Chain Fatty Acid (C26:25OH) Linked to the Lipid A Is Important for the Fitness of the Photosynthetic Bradyrhizobium Strain ORS278 and the Establishment of a Successful Symbiosis with Aeschynomene Legumes
Source: Front Microbiol. 2017 Sep 21;8:1821. doi: 10.3389/fmicb.2017.01821 (PMC5613085; doi:10.3389/fmicb.2017.01821)

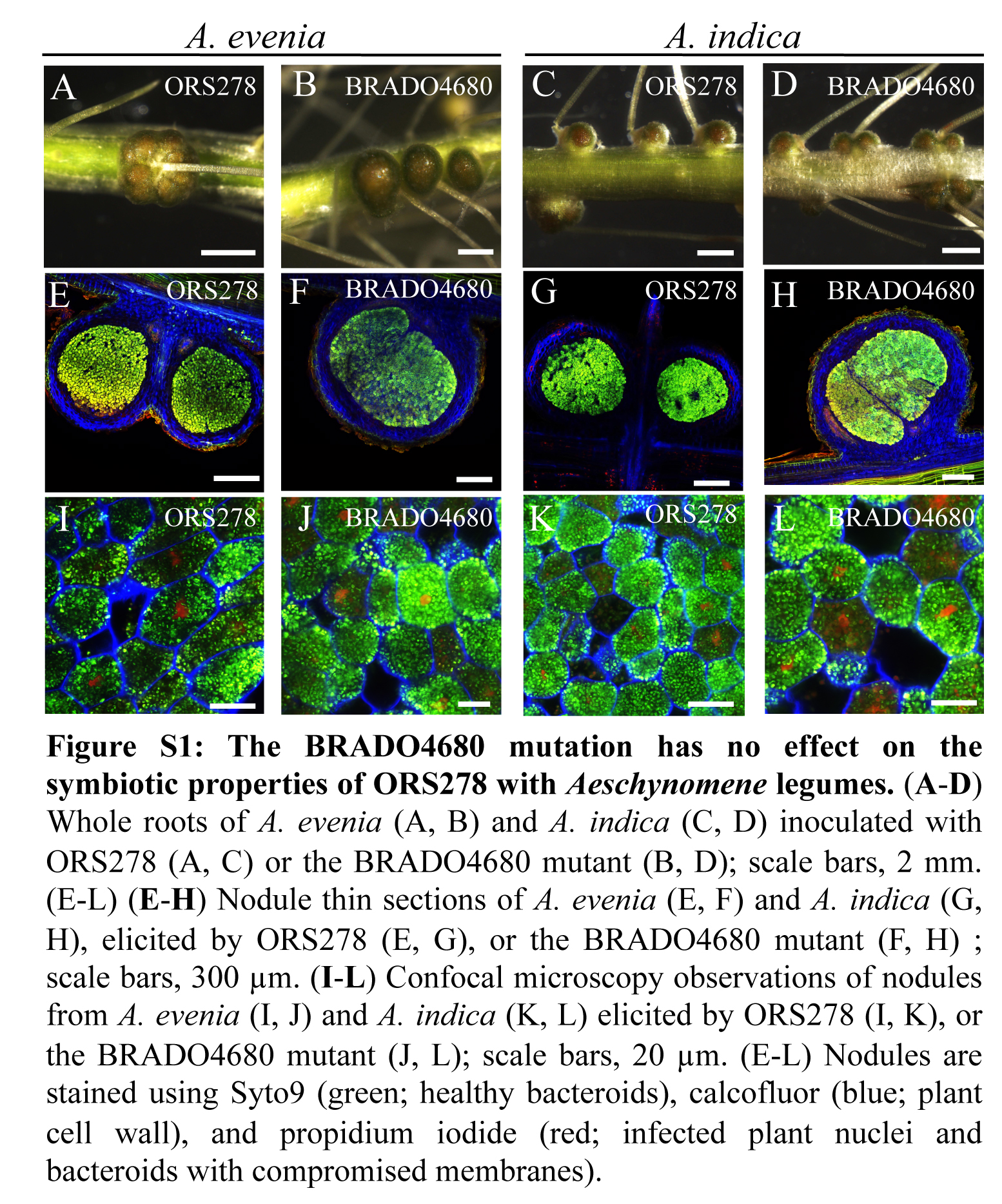

Supplement: Supplementary file 2 [file Image_1.JPEG]

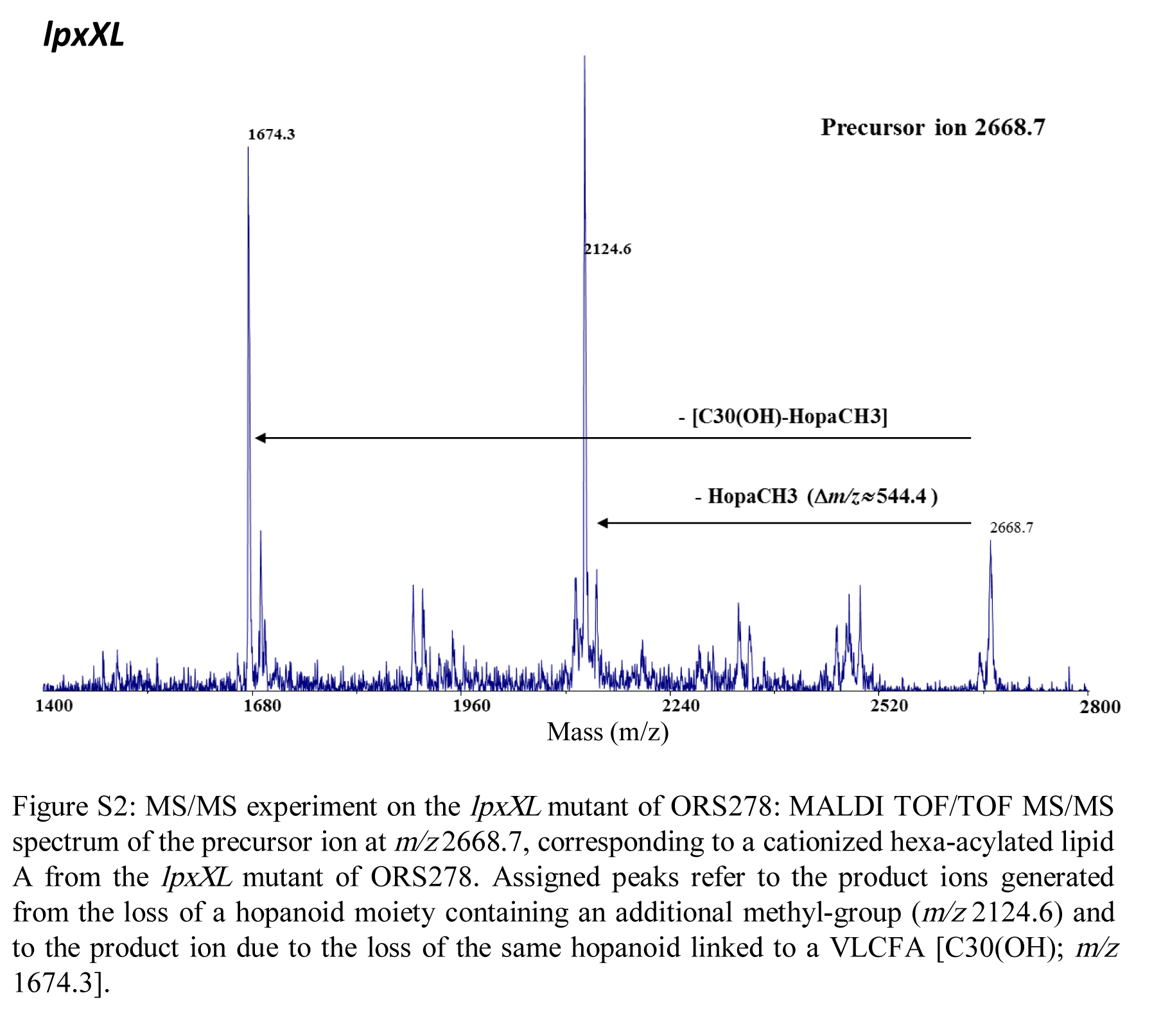

Supplement: Supplementary file 3 [file Image_2.TIF]

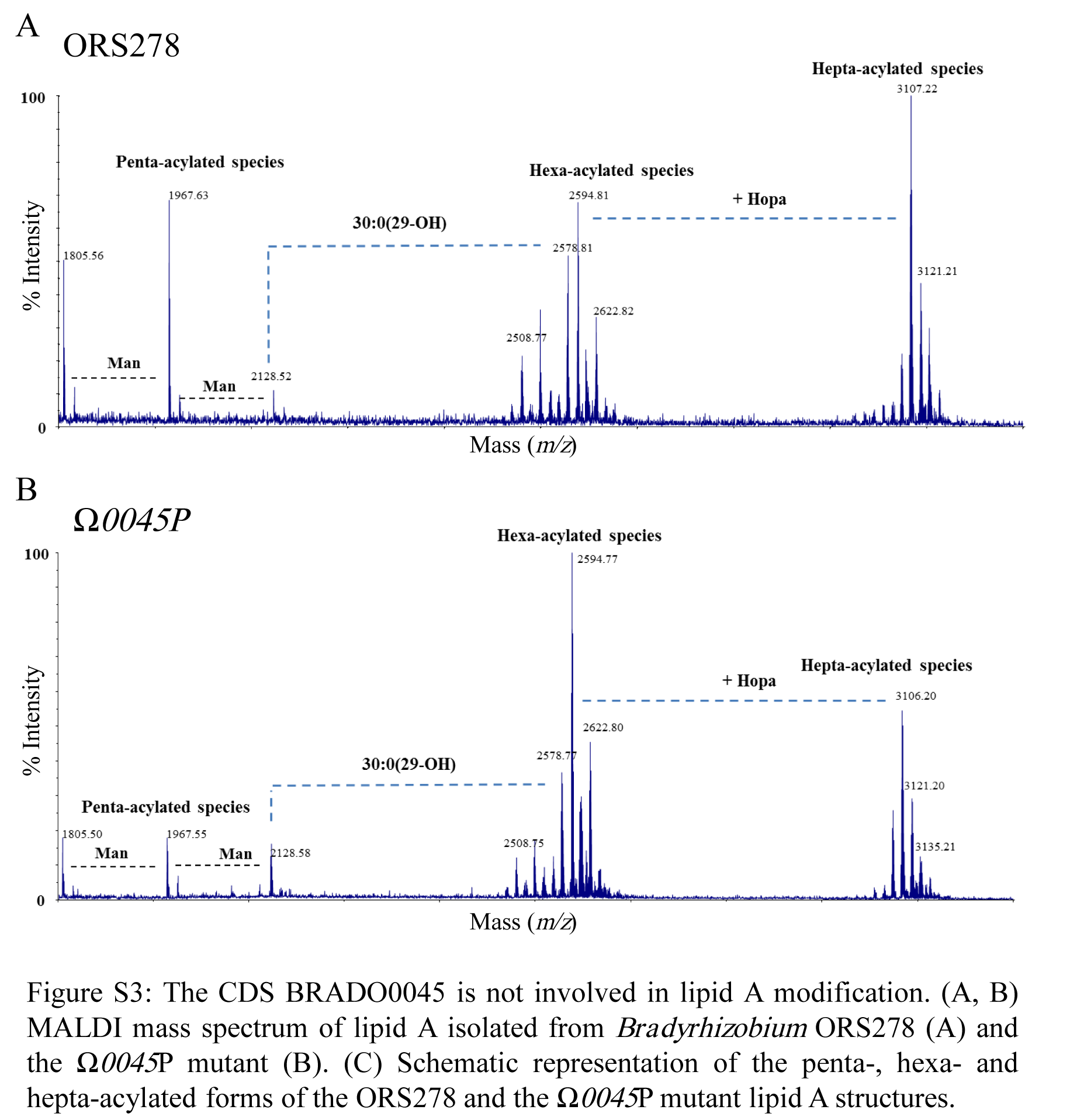

Supplement: Supplementary file 4 [file Image_3.TIF]

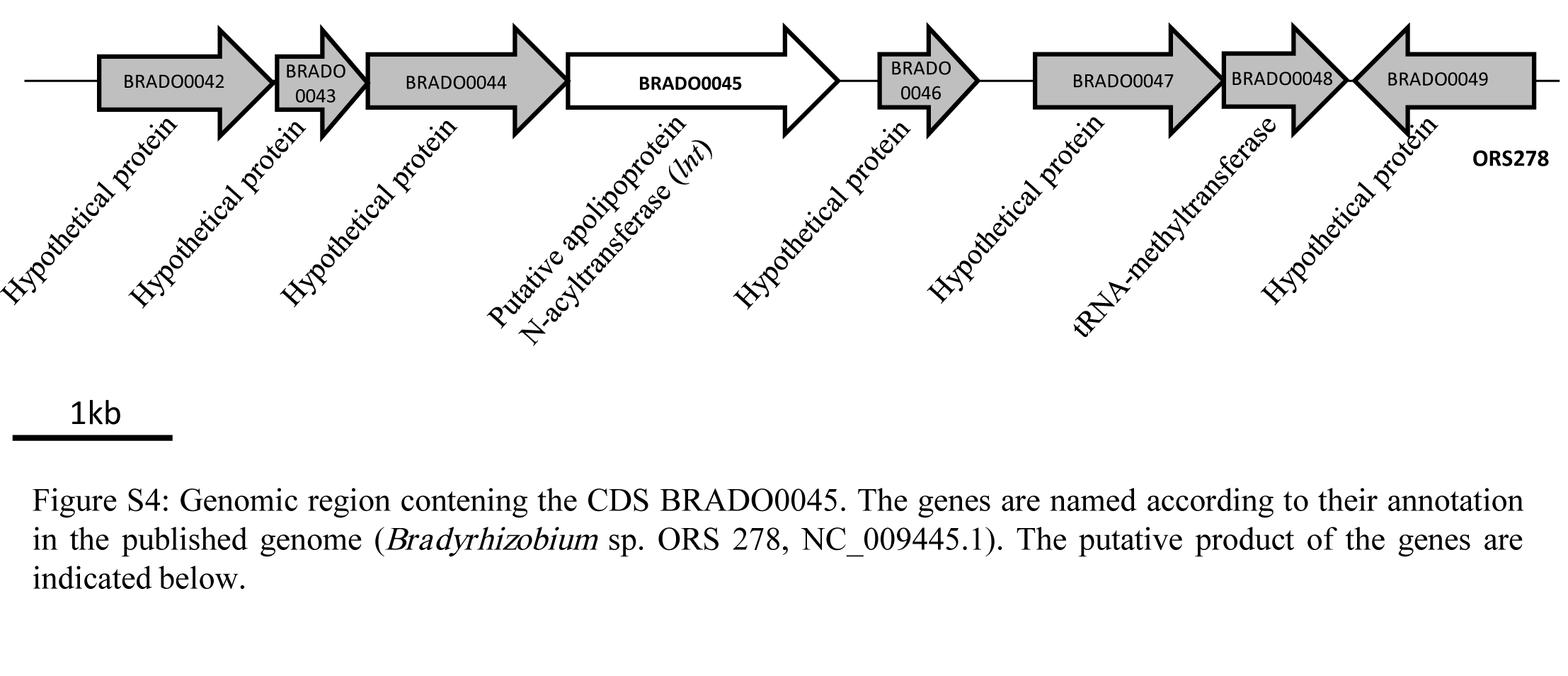

Supplement: Supplementary file 5 [file Image_4.TIF]
